# Viewing of Internet-Based Sexually Explicit Media as a Risk Factor for Condomless Anal Sex among Men Who Have Sex with Men in Four U.S. Cities

**DOI:** 10.1371/journal.pone.0154439

**Published:** 2016-04-27

**Authors:** Eric W. Schrimshaw, Nadav Antebi-Gruszka, Martin J. Downing

**Affiliations:** 1 Department of Sociomedical Sciences, Mailman School of Public Health, Columbia University, New York, New York, United States of America; 2 Research and Evaluation, Public Health Solutions, Inc., New York, New York, United States of America; The University of New South Wales, AUSTRALIA

## Abstract

The last decade has seen a dramatic increase in the availability of sexually explicit media (SEM) on the Internet. Men who have sex with men (MSM) report near universal use of SEM. However, this widespread use of SEM among MSM may contribute to more condomless anal sex. To examine the association of viewing SEM on the Internet and the number of condomless anal sex encounters among MSM, in 2012, an online survey was conducted of 265 MSM from New York, Philadelphia, Baltimore, or Washington D.C. who reported viewing SEM online in the past 3 months. Analyses were performed using negative binomial regression. Nearly all men reported viewing SEM featuring anal sex with (91%) or without (92%) condoms in the past 3 months. Neither viewing more hours of SEM per week or compulsively viewing SEM were associated with more condomless anal sex encounters. Rather, viewing a greater proportion of SEM containing condomless anal sex was associated with engaging in more condomless anal encounters (IRR = 1.25), while viewing a greater proportion of SEM containing anal sex where condoms were used was associated with fewer condomless anal sex encounters (IRR = 0.62). MSM reported that viewing SEM caused changes in their sexual fantasies, desires, and behaviors. These findings provide important insights for health policy and the design of interventions addressing SEM and condomless sex among MSM. The findings suggest that condom use by SEM performers may benefit not only actor health, but also have health implications for SEM viewers.

## Introduction

The greater availability of sexually explicit media (SEM) made possible by the Internet [1,2] has resulted in increased viewing of SEM. Data from the General Social Survey have documented a steady increase in SEM viewing among U.S. men since the 1970s [3]. The increased access to SEM may have had the greatest impact on men who have sex with men (MSM). Recent studies have found that nearly all MSM report viewing SEM, and that the vast majority (96–98%) report viewing it on the Internet [4,5].

Although SEM may have positive effects on the sexual lives of MSM [6], widespread use may have potentially adverse consequences. The proliferation of Internet-based SEM coincides with reported decreases in safer sex practices among MSM [7]. Behavioral theories, such as social cognitive theory [8], would suggest that viewing condomless anal sex in SEM may lead some MSM to engage in similar activities. Analysis of the content of online SEM videos found that 34% of MSM videos depicted condomless anal sex; with an approximately equal percentage depicting anal sex with condoms [9]. Furthermore, MSM prefer to watch SEM containing condomless anal sex more than sex with condoms [4,10].

Despite discussion of the potential of SEM to encourage condomless anal sex among MSM [1,7], relatively little research has examined this hypothesized relationship. Although some research has found that viewing more SEM in general is associated with a greater likelihood of engaging in condomless anal sex [11], other research has found that more viewing of SEM containing condomless anal sex is associated with a greater likelihood of engaging in the same behavior [5,10] and more condomless anal sex partners [4]. Given the small number of studies that have examined the role of SEM in condomless anal sex, additional research is greatly needed.

The past research has potential limitations. First, several studies have only examined whether or not participants engaged in any condomless anal sex, rather than the frequency of condomless anal encounters, thereby limiting the statistical power and underestimating the potential contribution of SEM to these behaviors. Several studies are also limited by the inclusion of a small number of potential (largely demographic) covariates; therefore, this work has identified few potential mechanisms by which SEM may contribute to condomless anal sex. Although the viewing of condomless anal sex in SEM has been previously examined, no prior studies have examined the converse–whether viewing SEM containing anal sex in which condoms were used is associated with greater condom use during anal sex. Finally, past studies have not examined whether MSM perceive SEM as contributing to less condom use among MSM, or whether MSM who engage in condomless sex seek SEM that contains such behaviors. Given these limitations, additional research is greatly needed.

The current paper reports on the results of an online survey of MSM who view SEM. It extends the prior research by examining whether viewing SEM (including total amount viewed, compulsive viewing, proportion containing condomless anal sex, and proportion containing anal sex with condoms) is associated with more condomless anal sex encounters and whether this association holds after imposing relevant covariates. In addition, to identify the potential causal direction between SEM and condomless sex, as well as potential mechanisms, additional questions were asked about participants’ perceptions of the effects of viewing SEM.

## Method

### Participants

This report is based on an online survey of 265 MSM who reported viewing Internet-based SEM in the past 3 months. Eligible men had to: (1) be 18 years of age or older; (2) report having had sex with another man in the past 12 months; (3) report having viewed male same-sex pornographic material on the Internet in the past 3 months; and (4) reside in the New York City, Philadelphia, Baltimore, or Washington, DC areas.

A total of 446 individuals consented to participate in the study. Of those, 132 (30%) screened out of the survey after not meeting eligibility criteria. An additional 42 (9%) surveys had missing data for questions of eligibility and therefore screened out of the study. Finally, 7 eligible surveys were identified as duplicate respondents based on identical IP addresses, followed by matching of participant characteristics. The duplicate surveys were removed resulting in a final sample of 265 participants for analysis.

### Procedure

Participants were recruited via advertisements on Craigslist and Facebook between June and November 2012. Elements of time-space sampling [12] were used to post study advertisements on Craigslist, a highly popular website used by MSM to meet sexual partners [13,14]. We used a random digit generator to select a one-hour increment of time, a geographic location, and a Craigslist category (i.e., men seeking men, casual encounters, or volunteers). Then, an advertisement was posted at the selected hour, in the appropriate city and category. Recruitment occurred twice a day between the hours of 8:00 am and 12:00 am. Study advertisements instructed anyone interested in participating to reply and request a link to the Internet survey, which was immediately provided through an automated response from a study e-mail account.

To target a broader audience of MSM, Facebook advertisements were also employed. Facebook is a highly efficient and cost-effective method of recruiting MSM for surveys [15,16]. The Facebook advertisements targeted users who were at least 18 years of age, identified as male, reported in their profiles that they were “interested in men,” and were within 50 miles of the four geographic locations. Individuals who clicked on the study advertisement were taken directly to the online survey.

Individuals accessing the online survey were prompted to review a consent form that outlined the study purpose and informed potential participants that upon completion of the survey there would be an opportunity to enter a random drawing for a $100 Amazon gift card. The study protocol received approval from the Institutional Review Board of Columbia University Medical Center.

### Measures

The online survey included a set of demographic questions to elicit participants’ age, race and ethnicity, sexual identity, HIV status, city of residence, and relationship status. Participants were asked to report the number of times they had anal sex with a man in the past 3 months. For those who reported anal sex, a follow-up question assessed how many of those encounters did not include a condom.

To measure time spent online watching SEM, participants were asked to report the number of hours spent viewing “man on man” pornography on the Internet in a typical week. Participants were asked to report the proportion of “man on man” Internet pornography they viewed that featured anal sex with a condom, as well as anal sex without a condom. Responses were scored on a 6-point scale: none, 1–24% (a little), 25–49% (some), 50–74% (much), 75–99% (most), or 100% (all). Unlike some past research [4], we measured and analyzed the proportion of SEM viewed that contained anal sex with a condom and anal sex without a condom as two separate variables (rather than a single continuous variable) because many MSM may view SEM that contains neither behavior (e.g., oral sex, masturbation only). Compulsive use of Internet websites to watch “man on man” pornography was assessed using a modified version of the Compulsive Internet Use Scale [17]. Participants also completed the Sexual Sensation Seeking scale [18].

To examine perceived effects of viewing SEM and potential mechanisms by which SEM may lead to sexual behaviors, men were asked to indicate for the past 3 months how often (1) they fantasized about engaging in similar acts they viewed in “man on man” pornography on the Internet, (2) viewing Internet pornography influenced the kind of sexual activity they desired, (3) viewing “man on man” pornography on the Internet led them to seek out sex afterward, (4) they acted out with another man any of the behaviors they viewed in “man on man” pornography, (5) viewing pornography on the Internet contributed to their engaging in risky sex, and (6) they had anal sex without a condom within hours of viewing SEM. Items were scored on a 5-point scale (*none of the time–every time*). Participants were also asked 2 items to gauge their perception of how much viewing Internet pornography contributes to them (and to other MSM) engaging in “riskier sex.” Response options were scored on a 5-point scale *(none–a lot*).

### Data Analysis

Descriptive statistics were computed for all variables. Because condomless anal sex was assessed as a count of the number of condomless anal encounters, we modeled this behavior using negative binomial regression [19]. Predictor variables included the proportions of Internet SEM viewed in the past 3 months that featured condomless anal sex and anal sex with condoms. Potential covariates included age, race/ethnicity, HIV status, relationship status, sexual sensation seeking, the number of hours spent viewing SEM on the Internet, and compulsive online SEM use.

## Results

Characteristics of the sample are presented in Table 1. Participants were primarily White/Caucasian (77%), self-identified as gay/homosexual (81.5%), and reported an HIV-negative status (90.2%). Two-thirds (67%) of the sample was recruited through Facebook, and approximately half of the men reported living in the New York City area (49.1%). Participants viewed, on average, five hours of SEM online per week, engaged in condomless anal sex more than seven times in the past 3 months with an average of 4 partners (S1 Dataset).

**Table 1:** Participant Characteristics (N = 265).

	M (SD) or n (%)
Age (Mean years)	32.9 (12.5)
Race/ethnicity	
White/Caucasian	204 (77.0)
Black/African American	14 (5.3)
Hispanic/Latino	21 (7.9)
Asian/Pacific Islander	14 (5.3)
More than one race/Other	12 (4.5)
Sexual identity	
Gay/Homosexual	216 (81.5)
Bisexual	39 (14.7)
Straight/Heterosexual	9 (3.4)
Queer	1 (0.4)
Relationship status	
Single	140 (52.8)
Relationship with a man	103 (38.9)
Relationship with a woman	22 (8.3)
HIV status	
HIV-negative	194 (73.2)
HIV-positive	21 (7.9)
Untested	32 (12.1)
Missing	18 (6.8)
City of residence	
New York	130 (49.1)
Philadelphia	45 (17.0)
Baltimore	17 (6.4)
Washington, DC	73 (27.5)
Mean sexual sensation seeking	2.8 (0.5)
Mean number of hours spent viewing SEM in a typical week	5.0 (6.6)
Mean compulsive Internet SEM use	1.04 (.80)
Mean number of condomless anal sex encounters in past 3 months (n = 187)	7.3 (13.5)
Mean number of male partners in past 3 months	4.0 (5.8)

### Viewing SEM and Number of Condomless Anal Encounters

Nearly everyone reported viewing any SEM online in the past 3 months that featured anal sex in which a condom was used (91.3%) or was not used (92%). We examined the hypothesis that the type of behaviors viewed in online SEM would be associated with more condomless anal sex encounters during the past 3 months. As shown in Table 2, in multivariate analyses controlling for all of the potential covariates, the number of condomless anal sex encounters increased by approximately 25% for every one unit increase in the proportion of SEM viewed online that featured condomless anal sex. In contrast, the number of condomless anal encounters decreased by approximately 38% for every one unit increase in the proportion of SEM viewed online that featured anal sex with condoms.

**Table 2:** Negative Binomial Regression: Number of Condomless Anal Sex Encounters by SEM Use and Covariates.

	Bivariate analysis (N = 187)		Multivariate analysis^c^ (N = 169)	
	IRR	95% CI	IRR	95% CI
Age	.97***	.95 –.98	1.00	.98–1.01
Recruited from Facebook^a^	.28***	.19 –.39	.36***	.23 –.58
Relationship with a woman^b^	1.96	.82–4.72	6.18***	2.25–16.98
Relationship with a man^b^	7.06***	5.05–9.88	10.28***	6.83–15.46
Sexual sensation seeking	1.30	.98–1.71	2.20***	1.46–3.30
Hours spent viewing SEM per week	1.02*	1.01–1.04	.98	.95–1.01
Compulsive Internet SEM use	1.02	.70–1.47	.90	.69–1.19
Proportion of SEM featuring anal sex with condoms	.70***	.61 –.82	.62***	.53 –.72
Proportion of SEM featuring condomless anal sex	1.28***	1.13–1.45	1.25**	1.06–1.47

Note: Bivariate and multivariate models excluded 74 men who reported no anal sex in the past 3 months and 4 additional men who failed to complete the anal sex questions. HIV status and race/ethnicity were not significantly associated with the outcome at either the bivariate or multivariate level and were removed from the model.

^a^Compared to those recruited from Craigslist.

^b^Compared to single men.

^c^Model χ^2^ = 201.14, *df* = 9, *p* < .001.

***p < .001

**p < .01

*p < .05

These associations between the proportion of behaviors viewed in SEM and number of condomless anal sex encounters were significant even after accounting for a number of covariates. For example, both the number of hours spent viewing SEM online and more compulsive use of online SEM were unrelated to the number of condomless encounters. Although sensation seeking, recruitment source (i.e., Craigslist vs. Facebook), and relationship status were associated with more condomless encounters, the proportions of SEM viewed featuring condomless anal sex and condomless anal sex encounters remained significant. Age, race/ethnicity, and HIV status (i.e., HIV-positive vs. negative/untested) were not significantly associated with more condomless anal encounters.

### Perceived Influence of Viewing SEM

To gain additional insights into the potential causal direction between viewing SEM and sexual behaviors, as well as to identify potential mechanisms by which viewing SEM may contribute to sexual behavior, men were asked to report on how much influence SEM had on their behavior and how often viewing SEM resulted in changes in behavior, fantasies, or desires (Table 3). Nearly all men reported that SEM contributed to other MSM engaging in “riskier sex” and nearly half reported that SEM had contributed to engaging in “riskier sex” themselves. Most men reported that viewing SEM had influenced their sexual desires, led them to fantasize about behaviors they had viewed in SEM, and had led them to seek out sex after viewing SEM in the past 3 months. Nearly a quarter (23%) reported engaging in condomless anal sex immediately after viewing SEM in the past 3 months.

**Table 3:** Perceptions of the Influence of SEM (N = 265).

	Percent Agreement	Mean (SD)
*Perceived Influence of SEM on Sexual Behaviors*		
How much has viewing SEM on Internet contributed to other MSM engaging in riskier sex	91%	2.7 (1.0)
How much has viewing SEM on Internet contributed to your engaging in riskier sex	48%	1.8 (1.0)
In past 3 months, how often has watching SEM on Internet contributed to you engaging in risky sex	29%	1.5 (1.0)
In the past 3 months, how often have you had anal sex without a condom within hours of viewing SEM on Internet	23%	1.4 (0.9)
*Perceived Mechanisms of SEM on Sexual Behavior (in past 3 months)*		
How often watching SEM on Internet influenced the kind of sexual activity you desired	83%	2.5 (1.1)
How often fantasized about engaging in acts similar to those in SEM on Internet	93%	3.2 (1.2)
How often acted out with another man things you viewed in SEM on Internet	70%	2.1 (1.0)
How often has viewing SEM on Internet led you to see out sex afterwards	55%	1.8 (0.9)

Perceived influence means are based on a 5-point scale ranging from *1* (None) to *5* (A lot). Frequency means are based on a 5-point scale ranging from *1* (None of the time) to *5* (Every time). Percent agreement is based on number who reported scores of 2 or more (A little–A lot; Some of the time–Every time).

## Discussion

This study provides insight into the understudied association between viewing condomless anal sex in SEM and engaging in more condomless anal sex encounters among MSM. The current findings extend the research in this area by examining whether SEM viewing is associated with more condomless anal sex encounters (rather than a dichotomous/binary variable of condomless anal sex), especially after controlling for multiple potential confounds. In addition, the study assessed perceived influence of SEM and identified potential mechanisms by which SEM viewing may influence sexual behavior.

Contrary to a frequent hypothesis in the field [4,11], we found that viewing more hours of SEM was not associated with more condomless encounters among MSM, thereby providing further evidence against this hypothesis [4]. Furthermore, we found that compulsive use of online SEM was also not associated with more condomless encounters. Taken together, these two findings make clear that it is not frequent viewing of SEM in general that is associated with condomless anal sex. Although some have advocated for restricting access to SEM [20,21], these findings suggest that policies to limit access or reduce the viewing of all SEM are unnecessary to prevent condomless anal sex. They also suggest that MSM who view SEM frequently or compulsively do not necessarily engage in more condomless anal sex. Thus, interventions may not be necessary to address frequent or compulsive SEM use for the purposes of reducing condomless sex.

Rather than viewing SEM in general, we found that it was the specific types of behaviors viewed in SEM that were associated with condomless anal encounters. Viewing a greater proportion of SEM that featured condomless anal sex was associated with a higher number of condomless anal sex encounters. Of critical interest here is that it was not the viewing of any condomless anal sex in SEM that was associated with condomless behaviors. Given that nearly all MSM (91%) reported viewing such behavior in SEM, such universal viewing of this behavior is unlikely to distinguish between those who engage in or do not engage in condomless behaviors. This indicates that regardless of how often MSM view SEM, the proportion of condomless anal sex viewed appears to be critical to behavior. As such, it is not the chronic or compulsive use of condomless SEM that is associated with condomless sex, but even casual viewing of condomless anal sex was associated with more engagement in condomless behaviors if it was a significant proportion of the SEM that participants viewed. Given that condomless anal sex and anal sex with condoms are portrayed with nearly equal frequency online [9], our findings, together with those of others [4,5,10], suggest that the availability of SEM online that features condomless anal sex may contribute to condomless anal sex among MSM viewers.

In addition, we found that viewing a greater proportion of SEM in which performers used condoms during anal sex was associated with fewer condomless anal sex encounters. This suggests that MSM who view more SEM containing condoms would be expected to use condoms more consistently. By assessing the proportion of SEM in which condoms were used separately from SEM in which condoms were not used, we were uniquely able to identify this positive association. However, as with viewing SEM containing condomless anal sex, it is the proportion viewed that includes condom use that is associated with fewer condomless sexual encounters. This positive association suggests that calls for restrictions on access to all SEM [20,21] could result in increased condomless behavior by eliminating this type of SEM. Our findings lend support to the argument for greater availability of SEM that contains anal sex in which condoms are clearly used by performers.

Although correlational design cannot determine whether MSM are influenced by behaviors they view in SEM or whether MSM choose to view SEM that contains behaviors they already enjoy, our findings do offer some insights into this issue. Specifically, questions were asked to what extent men believed that viewing SEM had influenced or changed their behaviors. Nearly half of MSM reported that viewing SEM had contributed to their engaging in “riskier sex.” Furthermore, 29% believed it has contributed to their engaging in “risky sex” in the past 3 months. These findings contradict those of some past research [6,22] that found MSM downplayed the contribution of SEM on their own sexual behaviors. Our findings similarly suggest potential mechanisms or pathways by which SEM could influence sexual behaviors. Specifically, the majority of participants agreed that SEM had influenced the types of sexual behaviors they desire, led them to fantasize about engaging in behaviors similar to those viewed in SEM, acted out behaviors viewed in SEM, and led them to seek out sex immediately after viewing SEM. Although men’s perception that SEM alters their behavior may not mean that SEM actually caused changes in behavior, it does offer additional evidence that MSM perceive SEM as having led to changes in their own behaviors, fantasies, and desires, and contribute to sexual behaviors among other MSM (rather than being merely a reflection of existing desires and behaviors). The validity of these perceptions is partially corroborated by recent findings that found that MSM who believed that condomless SEM influenced their condom use desires and norms were significantly more likely to engage in condomless anal sex and serodiscordant condomless anal sex [23].

These findings have implications for the SEM industry and public health efforts to regulate condom use in the industry. Condom use within the SEM industry has historically been self-imposed for the purpose of HIV/STD transmission prevention of SEM performers. However, there has been a significant increase in the prevalence of condomless anal sex in Internet-based SEM [9]. Thus, the Los Angeles City Council [24] passed regulations mandating condom use among adult film performers for the purposes of occupational safety and a similar ballot initiative will be voted on statewide in California in November 2016 [25]. Even more extreme, a bill has been introduced to the State Assembly of Utah that would declare all SEM (regardless of condomless content) a “public health crisis” [26]. Our findings offer empirical evidence to inform policy makers seeking to regulate the SEM industry. First, our findings suggest that regulation or limiting access to all types of SEM is unnecessary and unlikely to result in increased condom use. In fact, a reduction in SEM in which condoms are used may result in decreased condom use. However, our finding that those who view a greater proportion of SEM containing condomless anal sex engage in more condomless anal sex encounters suggests that strategies to promote condom use in the SEM industry may have benefits. Although condom use regulations have frequently been focused on reducing occupational HIV transmission [27], our findings suggest that such regulations could potentially be beneficial for the viewers of SEM by increasing the proportion of SEM that includes condom use. The need for condom use regulations may not be reduced by the use of frequent HIV testing or pre-exposure prophylaxis (PrEP) by SEM performers, as this will still result in consumers viewing SEM containing condomless anal sex. Without knowing that SEM performers are using PrEP, this may contribute to increased engagement in similar condomless behaviors among MSM viewers. Despite the potential benefits of increasing condom use within SEM (for both performers and viewers) by state or municipal governments, the potential efficacy of such regulations for reducing the availability of condomless anal sex in SEM are unclear. Regulations may lead the SEM industry to relocate to places without such regulations (including overseas locations). Such relocation may have undesirable consequences for extant industry practices such as frequent HIV testing (although the limited effectiveness of frequent testing must be also acknowledged) [27]. Alternatively, our findings provide information that may be persuasive and useful to SEM producers, websites who distribute SEM, and other venues where SEM is shown (e.g., gay bars, bathhouses, etc) to make the voluntary decision to produce, distribute, or show SEM that contains condom use. These findings also provide information for individual consumers of SEM (especially those who are concerned about their own temptations to engage in condomless anal sex) to make an informed decision about the types of SEM they choose to view. Future research on whether individuals in the SEM industry or consumers themselves would be willing to produce, show, or view more SEM containing condom use (and reasons against doing so) are needed to more fully inform the likelihood of such efforts being successful.

The study does have several limitations that require discussion. First, the study is based on a non-probability sample. Thus, self-selection and recruitment sources may limit the generalizability of these findings. However, the majority of adults use Facebook [28], and MSM are frequent users of websites like Craigslist for sexual partnering [13,14]. Further, this study sought MSM who view SEM online, therefore such online recruitment is logical [29]. Other limitations include that the sample is predominantly White, urban, and gay-identified; as is often the case in Internet-based research with MSM [14,30,31]. The study is also limited in that we were unable to examine whether viewing SEM would have different associations with condomless anal sex with regular partners or casual partners, serodiscordant or seroconcordant partners, or with insertive or receptive partners. Future research examining these more nuanced assessments of sexual behavior would be beneficial. Furthermore, the growing use of PrEP for HIV prevention that occurred since the data were collected may be a critical factor to account for in future research. Despite the relatively small sample, the use of a continuous outcome served to increase the statistical power and the significant findings make clear that the analyses were not underpowered. Finally, despite the addition of questions to assess the perceived influence of SEM on sexual fantasies, desires, and behaviors, the study was cross-sectional and therefore cannot determine the causal directionality between SEM and condom use. Future research using longitudinal methods (to address causal order) or qualitative methods (to gain insight into the experiences of MSM and how SEM influences their behaviors) would be greatly beneficial.

Despite these limitations, the findings offer critical insights into the role of viewing SEM on condomless anal sex among MSM. The study extends this research by documenting that it is not only the viewing of condomless anal sex in SEM that is associated with more condomless anal sex encounters, but that viewing anal sex with condoms is associated with fewer condomless encounters, even after controlling for potential covariates. Further, these findings offer insights into the causal direction and mechanisms by which SEM may contribute to sexual behaviors among MSM. As such, these findings offer multiple insights for the development of policy-level and behaviorally-based interventions to increase condom use among MSM.

## Supporting Information

S1 Dataset De-identified SEM Dataset. (SAV)
